# The boom and bust of the aphid’s essential amino acid metabolism across nymphal development

**DOI:** 10.1093/g3journal/jkab115

**Published:** 2021-04-08

**Authors:** Daniel Pers, Allison K Hansen

**Affiliations:** 1 Department of Entomology, University of California, Riverside, CA 92521, USA; 2 Department of Biochemistry, Vanderbilt University School of Medicine, Nashville, TN 37232, USA

**Keywords:** host-symbiont, aphid-*Buchnera*, development, DNA methylation, symbiosis, EvoDevo, essential amino acids

## Abstract

Within long-term symbioses, animals integrate their physiology and development with their symbiont. In a model nutritional mutualism, aphids harbor the endosymbiont, *Buchnera*, within specialized bacteriocyte cells. *Buchnera* synthesizes essential amino acids (EAAs) and vitamins for their host, which are lacking from the aphid’s plant sap diet. It is unclear if the aphid host differentially expresses aphid EAA metabolism pathways and genes that collaborate with *Buchnera* for the production of EAA and vitamins throughout nymphal development when feeding on plants. It is also unclear if aphid bacteriocytes are differentially methylated throughout aphid development as DNA methylation may play a role in gene regulation. By analyzing aphid gene expression, we determined that the bacteriocyte is metabolically more active in metabolizing *Buchnera*’s EAAs and vitamins early in nymphal development compared to intermediate or later immature and adult lifestages. The largest changes in aphid bacteriocyte gene expression, especially for aphid genes that collaborate with *Buchnera*, occurred during the 3rd to 4th instar transition. During this transition, there is a huge shift in the bacteriocyte from a high energy “nutrient-consuming state” to a “recovery and growth state” where patterning and signaling genes and pathways are upregulated and differentially methylated, and *de novo* methylation is reduced as evidenced by homogenous DNA methylation profiles after the 2nd instar. Moreover, bacteriocyte number increased and *Buchnera*’s titer decreased throughout aphid nymphal development. These data suggest in combination that bacteriocytes of older nymphal and adult lifestages depend less on the nutritional symbiosis compared to early nymphal lifestages.

## Introduction

Microbial symbionts allow animal hosts to thrive in niches that are otherwise uninhabitable. For example, symbionts can expand the metabolic potential of the animal by aiding in the digestion of food, and/or providing nutrient supplementation (McFall-Ngai *et al.* 2013; Flórez *et al.* 2015). Our understanding of how animals feed off of the metabolic products of microbial symbionts primarily resides from studies that examine a single developmental time point (Skidmore and Hansen 2017). Nevertheless, an animal’s energetic demands are known to fluctuate throughout development (Stockhoff 1993; Awmack and Leather 2002). By examining nutritional mutualisms through a developmental lens, a greater understanding can be gained in regard to how eukaryotic hosts integrate their physiology and development with their symbiont. Additionally, if the animal can regulate its symbiont’s production of nutrients throughout development, it can maximize its growth and development. Nevertheless, if nutritional symbionts are required to provide a specific level and composition of nutrients at specific developmental time points this can ultimately lead to increased dependence of the host on its obligate symbiont. Surprisingly, the mechanisms animals use to regulate symbiont provisioned nutrients throughout host development are unclear in most nutritional symbioses (McFall-Ngai *et al.* 2013).

The pea aphid, *Acyrthosiphon pisum*, and its bacterial endosymbiont, *Buchnera*, is an established model for nutritional symbioses. Aphids feed on phloem sap throughout development, a diet that lacks sufficient quantities of vitamins and essential amino acids (EAAs) for aphid growth and survival. *Buchnera* provides these essential nutrients to its aphid host, which the aphid cannot synthesize *de novo*, allowing the aphid to feed on this specialized plant niche (Shigenobu and Wilson 2011). *Buchnera* reside primarily in specialized aphid cells called bacteriocytes. Bacteriocyte cell number and cell size increase throughout nymphal development (Simonet *et al.* 2016; Colella *et al.* 2018). This rapid growth in bacteriocytes co-occurs with the high nutritional demand by the aphid as it grows, molts, and develops longer embryonic chains throughout nymphal development (Koga *et al.* 2012). *Buchnera* population growth rates can also be affected by development (Simonet *et al.* 2016; Zhang *et al.* 2019), in addition to aphid genotype (Chong and Moran 2016), nutritional status (Colella *et al.* 2018), and temperature (Humphreys and Douglas 1997; Dunbar *et al.* 2007; Moran and Yun 2015; Zhang *et al.* 2019).

Gene regulation in animals can be modified by epigenetic factors, such as DNA methylation (Romanoski *et al.* 2015). Aphids possess a full DNA methylation tool kit that may influence diverse processes (Walsh *et al.* 2010). For example, aphid DNA methylation is most strongly associated with the gain of pesticide resistance (Field 2000); however, additional studies have also proposed associations between DNA methylation and both wing dimorphism and sex-linked traits (Dixon 1977; Brisson 2010; Srinivasan and Brisson 2012; Pasquier *et al.* 2014; Mathers *et al.* 2019). Furthermore, a recent study identified a correlation between the differential expression and differential DNA methylation of critical amino acid metabolism genes within bacteriocyte tissue as *A. pisum* feeds on host plants that vary in amino acid profiles (Kim *et al.* 2018). DNA methylation is also critical for animal development as animals are known to experience an initial wave of erasing and remodeling of epigenetic marks (*i.e.* epigenetic reprogramming) during embryogenesis prior to differentiation (Ahuja and Issa 2000; Richardson 2003; Mallo and Alonso, 2013; however, see Xu *et al.* 2019). The role of DNA methylation in aphid development is unclear. Specifically, it is unknown how DNA methylation relates to bacteriocyte establishment and its fluctuations in both volume and symbiont titer throughout nymphal development (Douglas and Dixon 1987; Miura *et al.* 2003; Simonet *et al.* 2016). It is also unknown if epigenetic reprogramming continues as bacteriocytes develop and age.

Given these host and symbiont developmental changes, we predict that the aphid host requires different amounts of *Buchnera*’s EAAs throughout nymphal development. To our knowledge, no study has yet examined if the aphid host differentially expresses and methylates key aphid transporter and enzyme genes in bacteriocytes throughout nymphal development when feeding on plants. Ultimately, the profile and quantity of nutrients that immature aphid lifestages can obtain contributes to the reproductive output of the individual aphid (Wigglesworth 1960). Therefore, understanding how the aphid host metabolizes *Buchnera* produced EAAs and vitamins throughout aphid development will contribute to our evolutionary understanding of how aphid development has been shaped by its nutritional symbiosis with *Buchnera*. Moreover, by further identifying the patterns of DNA methylation in bacteriocytes a better understanding of epigenetics and its potential role in symbioses can be gained.

Here, we uncover new insights on how the aging nymphal to adult aphid host metabolizes EAAs and vitamins that are produced by *Buchnera* using RNAseq and whole-genome bisulfate sequencing (WGBS). Specifically, we identify major time points within bacteriocytes where there is a substantial increase and then decrease in the expression of aphid genes related to EAA biosynthesis and degradation, amino acid transport, and nitrogen recycling throughout nymphal development to the young adult lifestage. Additionally, we demonstrate that while the number of bacteriocytes increases throughout aphid development, symbiont titer decreases substantially after first instar. We also identify major developmental and epigenomic transitions that facilitate this massive shift in the aphid-*Buchnera* symbiosis and its nutrients.

## Materials and methods

### Aphid rearing and DNA/RNA extractions

Asexual clonal populations of the pea aphid, *A. pisum* (LSR1 strain), were reared on the 4–6 whorl stage of *Vicia faba* plants at 20°C with a 16:8-h light-dark cycle in Intellus Ultra controller Percival incubators (Percival Scientific, Inc., Perry, IA, USA) at conditions described in Pers and Hansen (2019). Three independent clonal sub-lines (Fava1, Fava2, and Fava3) were derived from a single female and then isolated, reared, and maintained for over 150 generations under these conditions.

Prior to the start of trials, host plant age and the relative number of aphid adults on each host plant were carefully monitored and controlled for more than four generations in order to reduce transgenerational effects caused by overcrowding and/or poor-quality hosts as described in Pers and Hansen (2019). Timed nymph lays of 0–30, 48–72, 96–120, 144–168, and 192–216 h were used to age nymphs to 1st, 2nd, 3rd, and 4th instar, and the pre-reproductive adult (referred to here as young adult) stages, respectively, as in Pers and Hansen (2019). Total molts shed were counted and the siphunculus length of a random subpopulation of aphids was measured for each instar to confirm the correct developmental life stage as described in Pers and Hansen (2019). Aphids were then briefly immobilized on ice before bacteriocytes were dissected out of aphid individuals for bacteriocyte counts, *Buchnera* titer quantification, WGBSseq, and RNAseq. See more details below and in Supplementary Materials and Methods.

### Bacteriocyte cell count and *Buchnera* titer quantification from bacteriocytes

For bacteriocyte counts, bacteriocytes from each individual aphid (*N* = 60 per lifestage, with 20 from each of 3 sublines) were counted at 5× magnification under a dissecting microscope to determine the average number of bacteriocytes per aphid at each lifestage. A one-way ANOVA and Tukey HSD multiple comparisons were used to determine significant (*P*-value ≤ 0.05) differences between lifestages.

*Buchnera* abundance or titer here is defined as the ratio of *Buchnera* single-copy genes/genomes to aphid single-copy genes/genomes, and is therefore an estimation of the number of *Buchnera* cells relative to aphid cells. We determined *Buchnera* titer with two independent approaches. For the first quantification approach, we used qPCR to amplify a single-copy *Buchnera* gene and a single-copy aphid gene from the same sample. The normalized ratio value (ave *Buchnera* DNA gene copy quantity/ave aphid DNA gene copy quantity) was computed for three biological replicates (sublines) for each lifestage using LinRegPCR (v.2020.0) (Ramakers *et al.* 2003; Ruijter *et al.* 2009).

In the second titer quantification approach, we used high-throughput sequencing data from the WGBSseq runs to compute the normalized ratio value (number of *Buchnera* reads mapped to the *Buchnera* genome/the number of aphid reads mapped to the aphid genome) for three biological replicates (sublines) for each lifestage. A one-way ANOVA and Tukey HSD multiple comparisons were used to determine significant (*P*-value ≤ 0.05) differences between lifestages for the normalized ratio values for both approaches. More details are provided in Supplementary Materials and Methods.

### Library construction and sequencing

For WGBSseq and RNAseq DNA and RNA, respectively, were co-extracted from the same bacteriocytes dissected from over 100 individual parthenogenetic aphids per subline from five developmental lifestages. DNA (WGBSseq) libraries were prepared from RNA-digested and bisulfite converted genomic DNA samples using EZ DNA Methylation Lightning Kit (Zymo, Irvine, CA, USA) and ACCEL-NGS Methyl-Seq Kit (Swift BioSciences, Ann Arbor, MI, USA) and sequenced on a single lane of the NovaSeqS4 platform (PE150). RNA libraries were prepared from poly-A purified RNA samples, after digestion of genomic DNA, using the KAPA Stranded mRNA-Seq Kit (Roche, Wilmington, MA, USA) and sequenced on a single lane of the Illumina HiSeq4000 platform (PE150) by the DNA Technologies and Expression Analysis Core at the UC Davis Genome Center. More details are provided in Supplementary Materials and Methods.

### Bioinformatic analyses of WGBSseq data

Raw WGBS reads were quality checked with FASTQC v.0.11.8 (Andrews 2010) and low-quality reads and adapter sequences were removed using Trimmomatic v.0.36 (Bolger *et al.* 2014). Trimmed reads were aligned against the *A. pisum* genome (RefSeq: 10963578; Li *et al.* 2019), the *Buchnera aphidicola* str. APS genome (RefSeq: 31108), and the *Escherichia* virus Lambda genome (RefSeq: 1534868) using Bowtie2 and Bismark (Krueger and Andrews 2011). Bismark and methylKit v1.10.0 Package within R (Akalin 2012) were used to calculate site-specific methylation data (read coverage and % methylation per base) for each sample. Hierarchical clustering analysis was used to visually analyze methylome profiles for each lifestage on methylKit. Profiles of differentially methylated sites (DMS) between two samples were obtained in methylKit by using logistic regression analysis to find CpG sites with greater than 25% methylation differences between lifestages (Akalin 2012; Kim *et al.* 2018). Statistical significance for each DMS was determined if the false discovery rate (FDR) corrected *P*-value was *q* ≤ 0.05. Additionally, methylKit was used to place each DMS into genomic context by annotating if each site overlapped with a genomic annotation of promoter, exon, intron, or intergenic regions of the DNA, and its distance from the nearest Transcription Start Site. More details are provided in Supplementary Materials and Methods.

### Bioinformatic analyses of RNAseq data

Raw RNAseq reads were quality checked with FASTQC v.0.11.8 (Andrews 2010) and low-quality reads and adapter sequences were removed using Trimmomatic v.0.36 (Bolger *et al.* 2014). Trimmed reads were aligned against both the *A. pisum* genome and the *Buchnera* genome (same genomes as above for WGBSseq) using HISAT2 v.2.1.0 (Kim *et al.* 2015). Off-target mapping was assessed using Centrifuge v1.0.3 (Kim *et al.* 2016). Reads, for each gene and transcript, which mapped to the *A. pisum* genome were quantified and assembled into transcriptomes using StringTie v.1.3.5 (Pertea *et al.* 2015). Abundance tables were generated for mapped genes and transcripts using Ballgown and were prepared for additional downstream analyses in R v3.6.0 using prepDE (Pertea *et al.* 2016). Reads were filtered to remove genes/transcripts with extremely low counts (min. 10 reads in one biological replicate), normalized (TMM), and genes/isoforms differentially expressed between lifestages were identified using EdgeR v3.26.6 and DEBrowser v1.12.3 using the exactTest (Robinson *et al.* 2010; Kucukural *et al.* 2019). Statistical significance was determined if the FDR-adjusted *P*-values were ≤0.01 and there was at least a twofold change in the normalized read count. Principal component analysis was used to qualitatively visualize normalized transcriptomic read profiles of each lifestage on DEBrowser and results can be found in Supplementary Figure S1. Gene Set Enrichment Analysis (GSEA, v4.0.0) (Subramanian *et al.* 2005) was used to determine which KEGG pathways were differentially regulated (normalized *P* ≤ 0.05 and FDR q ≤ 0.25), as described in Kim *et al.* (2018). Similar to Kim *et al.* (2018) to link DNA methylation patterns to gene expression patterns genes that were both differentially expressed and methylated significantly were identified for each comparison. More details are provided in Supplementary Materials and Methods.

### Data availability

Raw transcriptomic and methylomic data were uploaded to NCBI under SRA/BioProject accession number PRJNA613906 (Pers and Hansen 2020). Supplementary material is available on figshare: https://doi.org/10.25387/g3.14109851.

## Results

### Bacteriocyte numbers increase and *Buchnera* titer decreases as aphid nymphs age

**Figure 1:**
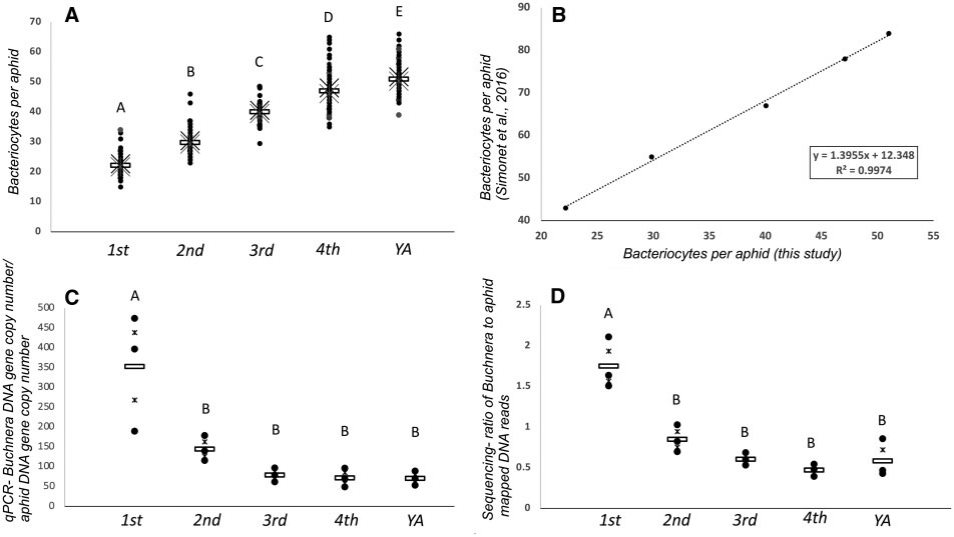
Phenotypic analyses of bacteriocyte samples from each aphid lifestage. Dissected bacteriocytes collected from aphids (*A. pisum*) of the following lifestages, 1st to 4th nymphal instars and the young adult (YA) lifestage. Letters above each sample represent samples that are significantly different from one another (*P* ≤ 0.05). For panels A, C, and D, the “x”s indicate the standard error (SE), each black dot represents a sample data point, and the horizontal open rectangle represents the average for the lifestage treatment. (A) The number of bacteriocytes identified from a single aphid for each lifestage based on dissections (*N* = 60 per lifestage, with 20 from each of 3 sublines). (B) Average number of bacteriocytes identified from a single aphid by Simonet *et al.* (2016) compared to the current study at each lifestage. (C and D) *Buchnera* relative abundance titer from each aphid lifestage (*N* = 3 samples per lifestage from each of the three sublines; each sample consists of the average of three technical replicates) determined by qPCR (C) and DNA high-throughput sequencing reads mapped to *Buchnera* and the aphid (D) (*N* = 3 samples per lifestage from each of the three sublines).

Here, we found that the total number of bacteriocytes significantly increase with age throughout nymphal and young adult development (Figure 1, A and B, Supplementary Table S1). We also found that *Buchnera* titer defined here as the normalized ratio value (average *Buchnera* DNA gene copy quantity/average aphid DNA gene copy quantity) was significantly higher in bacteriocytes during early nymphal development (1st) compared to the 2nd, 3rd, 4th, and the young adult lifestages using qPCR (Figure 1C). To validate these results using an independent approach, we examined *Buchnera* titer in bacteriocytes using a high-throughput DNA sequencing approach from dissected bacteriocytes collected in this study. We observed that the ratio of *Buchnera* to aphid mapped DNA reads throughout aphid development was significantly higher in bacteriocytes during early nymphal development (1st) compared to the 2nd, 3rd, 4th, and the young adult lifestages (Figure 1D, Supplementary Table S1). These results are in complete agreement with our qPCR results and suggest that *Buchnera* titer decreases in bacteriocytes as aphids age, especially after 1st instar. We observed a significant positive correlation of Pearson’s correlation of 0.862 (*P* < 0.000) between both *Buchnera* titer measurements from qPCR and high-throughput DNA sequencing.

### Aphid mRNA expression in bacteriocytes is dynamic across nymphal and young adult development

To determine how bacteriocyte gene expression changes throughout aphid development, RNA-seq transcriptomes were generated from aphid bacteriocyte tissue dissected from five lifestages (four nymphal and the young adult lifestage). On average, for each transcriptome sample, 12,661,852 high-quality reads were mapped to the target genome (*A. pisum*) and 3,940,040 high-quality reads were mapped to the symbiont’s genome (*Buchnera*), collectively accounting for approximately 83.5% of all high-quality reads (Supplementary Table S2, Supplementary Dataset S1). To further determine how bacteriocyte gene expression changes throughout aphid development, two separate lifestage comparisons were conducted (Table 1, Supplementary Datasets S2 and S3). First, we carried out a “sequential” gene expression analysis similar to Ginzburg *et al.* (2017) to identify changes in gene expression between two consecutive lifestages. For this comparison, gene expression of the prior lifestage is compared to the subsequent lifestage to understand how gene expression is different between sequential life stages, which is especially important in insect metamorphosis, incomplete or complete, because dramatic physiological and developmental changes may occur between insect molts. Second, similar to Yang *et al.* (2016), we performed a “lifestage specific” gene expression analysis to identify important stage-specific changes in gene expression compared to the average expression across the other lifestages (hereafter referred to as “compared to other stages”). This latter analysis was conducted to highlight important differentially expressed genes and pathways in bacteriocytes that are unique to a particular lifestage. When referring to RNAseq gene expression statistical analyses, we use the terms, “differentially expressed (DEGs),” “up-regulated,” and “down-regulated” to describe how mRNA abundance for a particular gene is expressed in one group compared to the other group. Only DEGs that are significantly upregulated or downregulated will be reported below. We define a DEG as significant if FDR-adjusted *P*-values are ≤0.01 and ≥ a twofold change in normalized read counts. When referring to individual DEGs, fold changes (FC) are reported as log_2_FC below, not simple FC. Subsequent analyses were also conducted on the pathway level using GSEA (Subramanian *et al.* 2005). Statistically significant changes in a pathway for one group are either “enriched,” “depleted,” or “not significantly enriched or depleted” compared to the other group in the comparison based on normalized *P* ≤ 0.05 and FDR *q* ≤ 0.25. All possible pair-wise comparisons for GSEAs and enrichment patterns of individual enzymes within pathways can be found in Supplementary Datasets S4 and S5.

**Table 1:** Number of significant differentially expressed^a^ genes (DEGs) or isoforms (DEIs), and differentially methylated^b^ sites (DMS) located on genes or isoforms from each sequential lifestage comparison^c^ and each lifestage-specific comparison^c^

	2nd *vs* 1st	3rd *vs* 2nd	4th *vs* 3rd	YA *vs* 4th	1st *vs* R	2nd *vs* R	3rd *vs* R	4th *vs* R	YA *vs* R
DEGs^a^	465	472	1,467	382	146	9	9	121	77
Upregulated genes^a^	94	133	415	363	82	0	0	21	64
Downregulated genes^a^	371	339	1,052	19	64	9	9	100	13
DEIs^a^	87	75	185	96	91	34	27	59	60
Upregulated isoforms^a^	28	43	81	63	76	24	24	27	55
Downregulated isoforms^a^	59	32	104	33	15	10	3	32	5
DMS^b^	763	2,722	2,243	1,173	1,841	644	291	199	220
Hypermethylated sites^b^	671	2196	695	589	85	324	269	162	180
Hypomethylated sites^b^	92	526	1548	584	1756	320	22	37	40
Differentially methylated and expressed genes^a^^,^^b^	9	13	87	3	5	0	0	2	0
Differentially methylated and expressed isoforms^a^^,^^b^	2	12	16	5	0	0	0	0	0

First to 4th and YA refer to nymphal instars and the young adult lifestage, respectively, of *A. pisum*; Rest (R) = average of all other lifestages; *N* = 3 biological replicates per lifestage for each comparison.

a FDR corrected *P*-value ≤ 0.01, normalized read count FC ≥ 2.0X.

b ≥25% change in methylation level, FDR-adjusted *P*-value ≤ 0.05, and minimum coverage of 10 reads.

c X *vs* Y indicates that X is compared to Y.

Sequential lifestage gene expression analysis uncovered a total of 2786 genes differentially expressed in bacteriocytes between sequential lifestage comparisons, with a majority of these differentially expressed genes occurring between the 3rd and 4th instar transition (Table 1, Supplementary Dataset S2). For each of these sequential lifestage comparisons, the majority of DEGs are downregulated in bacteriocytes as the aphid nymph develops, except between the 4th instar and the young adult stage, when 95% of DEGs are upregulated when the 4th instar nymph molts into an adult (Table 1). Lifestage-specific gene expression analysis found that most aphid genes in bacteriocytes are differentially expressed during the 1st instar (146 DEGs) followed by the 4th instar (121 DEGs) and the young adult stage (77 DEGs), when compared to other stages (Table 1, Supplementary Dataset S2). In contrast, the 2nd (9 DEGs) and the 3rd (9 DEGs) instars displayed the lowest number of stage-specific DEGs in bacteriocytes compared to other stages (Table 1, Supplementary Dataset S2).

**Figure 2:**
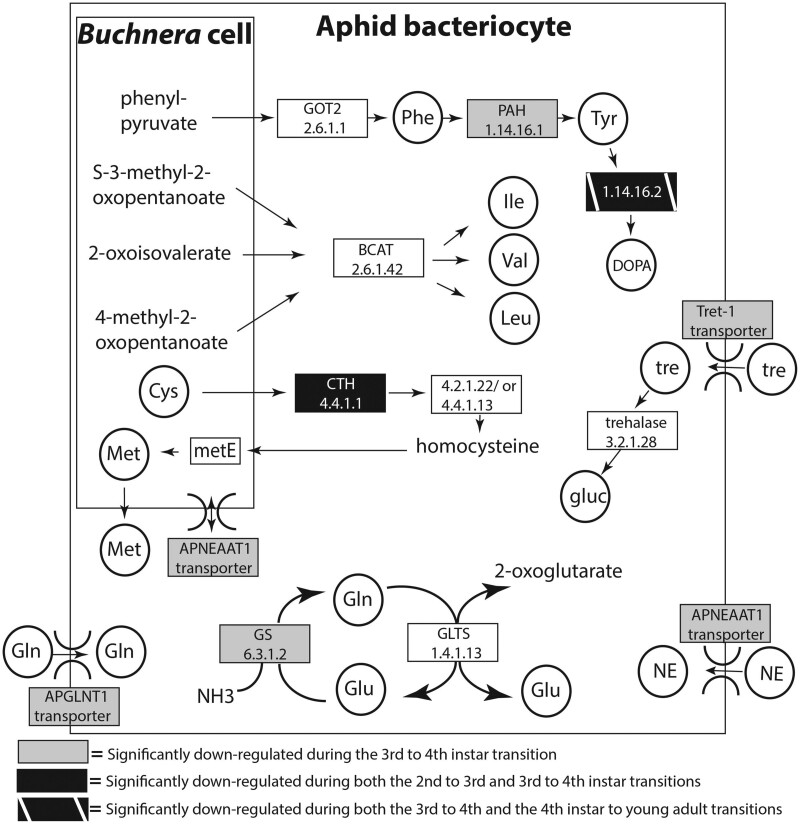
Differential expression of key collaborative aphid genes that regulate the integrated aphid-*Buchnera* amino acid metabolism across nymphal development. Gene boxes are annotated with either E.C. numbers and/or gene names. Gene boxes are shaded accordingly if the gene is significantly downregulated in bacteriocytes of one lifestage compared to another (*P* ≤ 0.01 and FC ≥ 2.0).

Of these DEGs, 46 are aphid genes that are potentially involved in the nutritional symbiosis with *Buchnera* based on previous bacteriocyte gene expression studies (Hansen and Moran 2011; Poliakov *et al.* 2011) (Supplementary Table S3, Supplementary Figure S2, Supplementary Dataset S6). For example, *Buchnera* depends on aphids for both intermediate and terminal steps in EAA biosynthesis (Figure 2; Wilson *et al.* 2010; Hansen and Moran 2011; Poliakov *et al.* 2011). These collaborative aphid genes include cystathionase-like enzyme (4.4.1.1), which is hypothesized to produce cystathionine from cysteine, an important intermediate for methionine biosynthesis by *Buchnera* (Wilson *et al.* 2010). Here, the cystathionase-like enzyme (4.4.1.1) is downregulated as the aphid ages for the sequential lifestage comparisons 2nd *vs* 3rd (log_2_FC = −1.5X) and 3rd *vs* 4th (log_2_FC = −2.3X) instars (Figure 2, Table 2, Supplementary Table S3, Supplementary Dataset S6). Two other collaborative aphid enzymes, aspartate transaminase (*GOT2*; EC 2.6.1.1) and phenylalanine hydroxylase (*PAH*; EC 1.14.16.1), are hypothesized to be important intermediates and terminal steps for *Buchnera*’s production of phenylalanine and tyrosine, respectively (Wilson *et al.* 2010; Figure 2). While *GOT2* (2.6.1.1) is not found to be differentially expressed following sequential lifestage or lifestage-specific gene expression analysis comparisons, *PAH* (1.14.16.1), is downregulated (log_2_FC = −2.3X) as the aphid ages in 3rd *vs* 4th instar transition (Figure 2, Table 2, Supplementary Figure S3, Supplementary Table S3, Supplementary Dataset S6). The production of dihydroxyphenylalanine (DOPA) from tyrosine via the aphid enzyme tyrosine 3-monooxygenase (1.14.16.2), which is a rate-limited step, is downregulated as the aphid ages in 3rd *vs* 4th instar transition (log_2_FC = −3.1X) and is downregulated further as the aphid ages in the 4th instar *vs* young adult lifestage (log_2_FC = −7.4X) transition (Figure 2, Supplementary Dataset S2).

**Table 2:** Aphid genes involved in the symbiotic relationship with *Buchnera* that are significantly^a^ differentially expressed between sequential lifestage comparisons^b^ within bacteriocytes that are involved in the amino acid metabolism and transport

Gene ID	Annotation	EC numbers	2nd *vs* 1st	3rd *vs* 2nd	4th *vs* 3rd	YA *vs* 4th
LOC100159667	*ApGLNT1*	N/A: Transporter			Down	
LOC100168251	*ApNEAAT1*	N/A: Transporter			Down	
LOC100159664	Active transporter; 2-oxoglutarate carrier	N/A: Transporter		Down	Down	
LOC100159441	Trehalose transporter; Tret1	N/A: Transporter			Down	
LOC100166971	Phenylalanine 4-monooxygenase; *PAH*	1.14.16.1			Down	
LOC100159560	Cystathionase-likeF	4.4.1.1		Down	Down	
LOC100160139	Glutamine synthetase 2; *GS2*	6.3.1.2			Down	
LOC100163589	Phosphoserine transaminase	2.6.1.52			Down	
LOC100161178	L-Threonine aldolase 2	4.1.2.48	Down			
LOC100160265	Asparagine synthase	6.3.5.4	Down	Down		
LOC100164179	Asparaginase	3.5.1.1; 3.1.1.5			Down	
LOC100160095	Asparaginase	3.5.1.121			Down	
LOC100167144	Asparaginase	3.5.1.121			Down	

a FDR corrected *P*-value ≤ 0.01, normalized read count FC ≥ 2.0X.

b X *vs* Y indicates that X is compared to Y. down = downregulated; empty cell = not significantly differentially expressed.

In addition to synthesizing metabolic precursors, intermediates, and products, another important function within the aphid-*Buchnera* symbiosis is the transport of these metabolites and waste products from where they are produced to where they are utilized. Aphids supply *Buchnera* with non-essential amino acids (NEAAs), such as glutamine and glutamate, via bacteriocyte transporters and by recycling ammonia in bacteriocytes to serve as the building blocks and amino donors in EAA biosynthesis (Figure 2; Hansen and Moran 2011; Price *et al.* 2014; Feng *et al.* 2019). Here, we found that the aphid enzyme glutamine synthetase (6.3.1.2), which is hypothesized to recycle waste ammonia into two glutamates (Glu) via the GOGAT cycle (Hansen and Moran 2011), is downregulated (log_2_FC= -1.8X) as the aphid ages in the 3rd *vs* 4th instar transition (Figure 2, Table 2, Supplementary Figure S3, Supplementary Table S3, Supplementary Dataset S6). In regard to aphid transporters, the glutamine transporter 1 (*ApGLNT1*), which imports glutamine (Gln) from the hemolymph into the bacteriocytes, and the NEAA transporter 1 (*ApNEAAT1*), which shuttles NEAAs from the aphid hemolymph to the bacteriocyte cytoplasm and between the bacteriocyte cytoplasm and symbiosomal space around *Buchnera* cells (Price *et al.* 2014; Feng *et al.* 2019), are both significantly downregulated as the aphid ages in 3rd *vs* 4th instar transition (log_2_FC = −1.9X and −2.0X, respectively; Figure 2, Table 2, Supplementary Figure S3, Supplementary Dataset S6). In addition, 25 additional transporters that were previously observed to be upregulated in bacteriocytes compared to body tissues during 4th instar (Hansen and Moran 2011; Kim *et al.* 2018) were significantly downregulated here in bacteriocytes as aphids age during the 2nd *vs* 3rd and/or the 3rd *vs* 4th instar transitions (Supplementary Table S3, Supplementary Dataset S6). One of these transporters, the facilitated trehalose transporter (Tret-1), is important in importing trehalose into bacteriocytes for its conversion by the aphid enzyme trehalase-like isoform X1 (3.2.1.28) into D-glucose, which *Buchnera* requires from the aphid for glycolysis. While the trehalase-like isoform X1 (3.2.1.28) is not found to be differentially expressed based on sequential lifestage or lifestage-specific gene expression analysis comparisons, the transporter Tret1 is significantly downregulated (log_2_FC = −1.5X) in bacteriocytes as the aphid ages in the 3rd *vs* 4th transition (Figure 2, Table 2, Supplementary Table S3, Supplementary Dataset S6).

Given that bacteriocytes are novel developmental outputs evolved to harbor endosymbiont bacteria and facilitate its symbiosis with the insect host, it is reasonable to hypothesize that a subset of developmental genes, modules, and/or pathways have been coopted to also regulate the symbiotic functions of these novel cells. In fact, a number of developmental genes have been demonstrated to mark bacteriocytes as they proliferate and migrate during development (Braendle *et al.* 2003). Here, 81 developmental genes were found to be differentially expressed between at least one lifestage comparison throughout nymphal development (Shigenobu *et al.* 2010; Supplementary Figures S4 and S5, Supplementary Table S4, Supplementary Dataset S6). Most of these DEGs were either upregulated during early to intermediate nymphal development (1st to 2nd or 2nd to 3rd instar transition) or downregulated during intermediate to late nymphal development (3rd to 4th instar transition; Supplementary Table S4, Supplementary Dataset S6). During the 3rd to 4th instar transition, when major symbiosis genes are downregulated as the aphid ages to 4th instar, two canonical hox genes (*abdominal-A*, *deformed*), 10 noncanonical homeodomain-containing genes (such as: *empty spiracles*, *mirror*, *prospero*, *tailup*, *twin of eyeless*), and dozens of signaling factors (mainly from the TGF-β and Wnt signaling pathways) are downregulated during this same instar transition (Supplementary Table S4, Supplementary Dataset S6).

### Bacteriocyte pathway profiles dynamically change across aphid development

**Figure 3:**
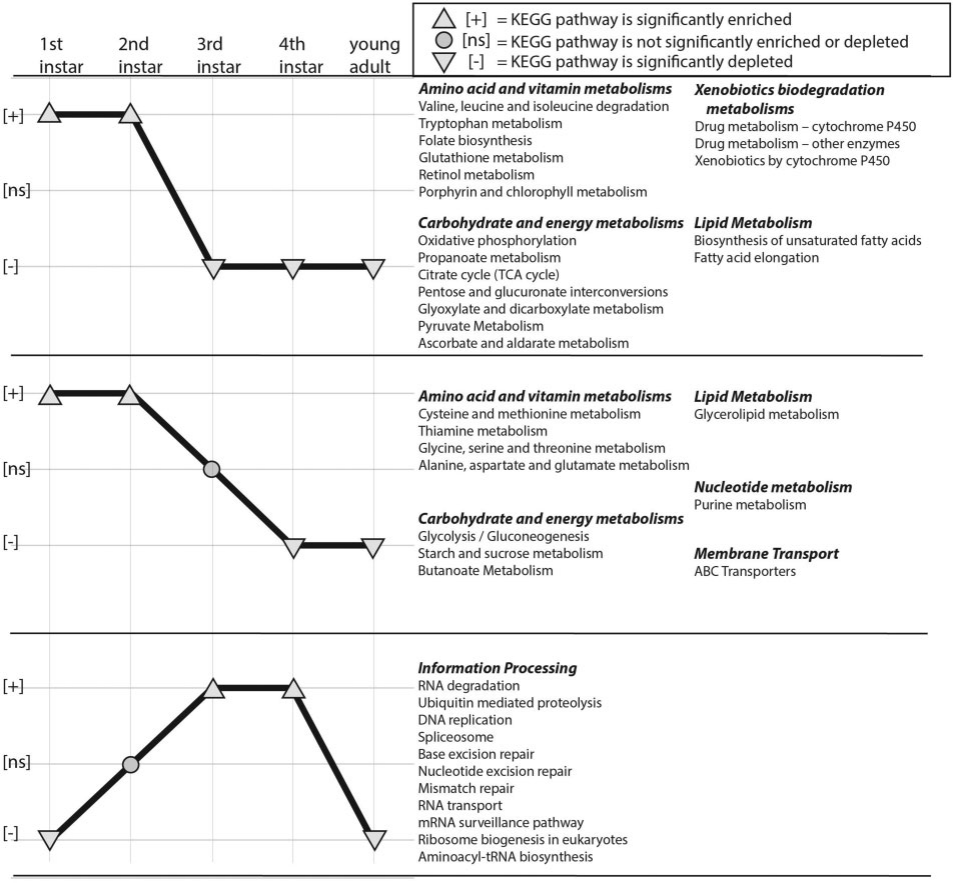
Co-expression patterns of KEGG pathways in bacteriocytes during aphid development. The *x*-axis represents aphid lifestages in chronological order from 1st instar to the young adult lifestage. The *y*-axis indicates three discreet states based on the significant statistical results from GSEA analyses for each lifestage compared to the average of other lifestage [*e.g.* positively enriched (+), not significantly enriched or depleted (ns), or negatively depleted (−)]. Enriched, nonsignificant, and depleted pathways are represented by an upward-facing arrowhead, circle, or a downward-facing arrowhead, respectively. Each panel represents KEGG pathways that display identical enrichment patterns throughout development.

**Figure 4:**
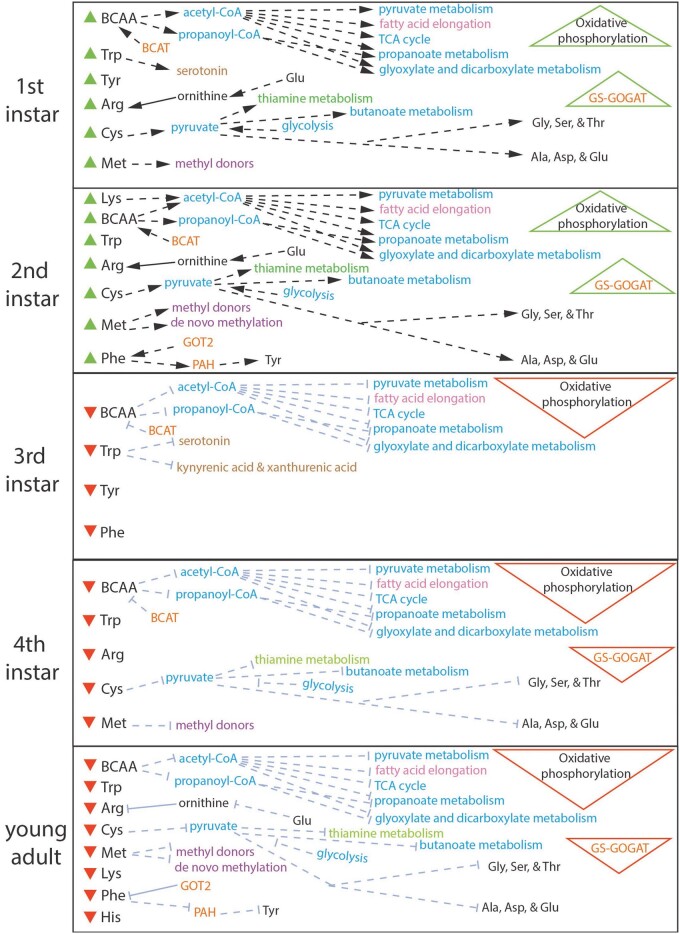
Metabolism of EAAs in aphid bacteriocytes throughout aphid nymphal development and the young adult lifestage in *A. pisum*. The metabolic pathways displayed are significantly enriched [represented by saturated text, black arrows (→), and green upward-facing arrowheads (Δ)] or depleted [represented by gray bar-headed lines (⊣), and red downward-facing arrowheads (∇)] for a particular lifestage compared to the average of other lifestages using GSEA. If pathways are presented in one lifestage panel but are absent from another then the pathway was not significantly enriched or depleted for that life stage. Text color represents specific GSEA metabolism categories: blue = carbohydrate metabolism, pink = lipid metabolism, green = metabolism of cofactors and vitamins, black = amino acids and amino donors, purple = methylation, brown = neurotransmitters and intermediates of ommochrome pathway, orange = aphid genes that are hypothesized to complement *Buchnera*’s EAA pathways or recycle ammonia for the production of amino donors for the biosynthesis of *Buchnera* produced EAA. Solid line = putative *Buchnera* produced metabolite; dashed lines = aphid produced metabolite/metabolism.

Examining aphid bacteriocyte mRNA expression profiles on the pathway level using GSEA, lifestage-specific comparisons reveal an overall pattern where the membrane transport, EAA, NEAAs, vitamin, carbohydrate and energy, xenobiotics biodegradation, lipid, and nucleotide metabolism KEGG pathways are enriched significantly in the 1st and 2nd instars and are depleted significantly as the aphid nymph ages in the 3rd, 4th, and the young adult stage compared to others (Figures 3 and 4, Supplementary Figure S6, Supplementary Datasets S4 and S5). Interestingly, subsets of specific EAA, NEAA, and vitamin pathways share identical patterns of enrichment and depletion for stage-specific comparisons (Figure 3, Supplementary Dataset S4, see Supplementary Results for details), suggesting that these latter pathways are either coregulated and/or are dependent on one another for amino donors and/or other intermediates. In contrast, 22 KEGG information processing pathways that are important for cell maintenance, repair, and/or recycling are all enriched in the bacteriocyte during 3rd and/or 4th instar lifestages compared to other stages (Figure 3, Supplementary Figure S7, Supplementary Dataset S4). These pathways appear to reach peak enrichment as the metabolism pathways become depleted, suggesting these two groups are independently or even antagonistically regulated. Finally, developmental and signaling pathways display no conserved pattern of enrichment/depletion within bacteriocytes across nymphal development (Supplementary Figure S7, see Supplementary Results for details). This dynamic pattern of enrichment across development and between pathways suggests that different pathways are necessary at different developmental time points.

### Aphid DNA methylation patterns in bacteriocytes are homogenous after 2nd instar when feeding on fava

To determine how methylation patterns change and how these changes may affect bacteriocyte gene expression throughout aphid development, DNA methylomes were generated from the same bacteriocyte tissue mRNA was isolated from for RNAseq above, which was dissected from five aphid lifestages (four nymphal and the young adult stage). On average, for each methylome sample, 45,017,179 high-quality reads mapped to the target *A. pisum* genome and 33,427,245 high-quality reads mapped to the *Buchnera* genome, accounting for approximately 52.2% of the total high-quality reads (Supplementary Table S5). A sufficient target genome coverage per sample of ∼25X was achieved (Ziller *et al.* 2015; Supplementary Table S5) with an average of 14.6X coverage per cytosine (Supplementary Table S6). The genome-wide percentage of aphid CpG methylation was on average ∼2.6% per sample after correcting for background levels of spiked in Lambda phage control DNA (Supplementary Materials and Methods). *Buchnera* and Lambda demonstrated similar background levels of CpG methylation, suggesting that *Buchnera* does not methylate its genome at CpG sites (Supplementary Tables S6 and S7).

Hierarchical clustering results reveal that early nymphal stages of bacteriocytes (1st and 2nd) exhibit heterogeneous methylation profiles between biological replicates and lifestages. In contrast, later lifestages (3rd, 4th, young adult) are highly homogenous in methylation profiles and do not exhibit lifestage-specific methylation profiles, as biological replicates of different lifestages cluster closely together (Supplementary Figure S8). Methylation data were analyzed using the same lifestage comparisons as for gene expression data. Sequential lifestage analysis of the % methylation of specific aphid CpG sites between lifestages reveals that ∼2X more significant differentially methylated CpG sites (DMS) occurred between the 2nd and 3rd instar stages compared to the average of DMS for the other lifestage transitions (Table 1, Supplementary Table S8, Supplementary Dataset S7). Lifestage-specific analyses revealed that ∼5.4X more DMSs occurred in the 1st instar compared to the average of DMS for the other lifestages. These results suggest that early nymphal instars are subject to large amounts of lifestage-specific DNA methylation changes, which was corroborated by Hierarchical clustering results above (Table 1, Supplementary Table S8, Supplementary Figure S8, Supplementary Dataset S7). The majority of these DMSs for both comparisons occurred within the gene bodies (*i.e.* exon and intron regions), similar to what was previously found in aphid bacteriocytes (Kim *et al.* 2018) and other insects in general (Glastad *et al.* 2011). Only about 15% of these DMSs overlapped with promoter regions (Supplementary Table S8).

To further identify candidate genes potentially regulated by DNA methylation in bacteriocytes throughout development, genes and splice isoforms that were both differentially expressed and methylated within each sequential and lifestage-specific comparison were identified (Supplementary Datasets S2, S33, S7, and S8). Overall, 112 DEGs that were also DMS within the same sequential lifestage comparison were identified (Table 1, Supplementary Dataset S8). The majority (78%) of these genes occurred during the 3rd to 4th instar transition, suggesting that changes in methylation may be linked to the differential regulation of these intermediate to late nymphal lifestage genes; although this may just be a sampling effect, because most genes were differentially expressed during this lifestage transition compared to others. Only seven DEGs were also DMS following lifestage-specific analyses, five and two within the 1st and 4th instars, respectively. Given that DNA methylation is known to be linked to not just gene expression patterns, but also differential splicing, differential expression of gene splice types was also investigated in a similar manner as DEGs. Overall, 35 of 443 differentially expressed isoforms (DEIs) and 0 of 271 DEIs were also DMS within the same sequential or lifestage-specific comparison, respectively (Table 1, Supplementary Datasets S3 and S8). Specifically, 3 out of 163 symbiotic genes and 12 out of 387 developmental genes were identified to be both significantly DMS and DEG during the same sequential or lifestage-specific comparisons (Supplementary Tables S3 and S4, Supplementary Dataset S6). For example, the three symbiotic genes that were both significantly DMS and DEG are a mitochondrial pyruvate carboxylase (6.4.1.1), a 5ʹ-nucleotidase (3.1.3.5), and an active transporter similar to a 2-oxoglutarate carrier protein. In regard to developmental genes, we found one of the eight canonical hox genes (*Ubx*), eight noncanonical homeodomain-containing genes, and dozens of signaling factors are differentially methylated (both hypo- and hypermethylation) within bacteriocytes during aphid larvalgenesis (Supplementary Dataset S6). Additionally, three of these differentially methylated hox genes and four of these differentially methylated signal factors are also differentially expressed during the same lifestage transition or the following lifestage transition (Supplementary Dataset S6).

## Discussion

During animal development, amino acids are not only incorporated into proteins, but they are also catabolized into metabolic intermediates such as neurotransmitters and nucleotides and can be degraded for the provision of energy. By analyzing aphid bacteriocyte mRNA expression patterns throughout aphid development, we determined that some of the collaborative aphid intermediate and terminal steps of EAA biosynthesis with *Buchnera* (Figure 2) in addition to the down-stream pathways that metabolize EAA into proteins, hormones, and energy were upregulated during the early nymphal lifestages (1st and 2nd instar) compared to intermediate or later lifestages (based on both sequential lifestage comparisons and when comparing a specific nymphal or adult lifestage to the average of other lifestages; *i.e.* stage specific) (Figures 2–4). Moreover, the regulation of *ApGLNT*, *ApNEAAT*, Tret-1, and 24 additional aphid transporter transcripts are downregulated during the 3rd to 4th instar of aphid development (Figure 2). These transporters play a role in the movement of NEAAs and sugars, which are required by *Buchnera* (Hansen and Moran 2011; Kim *et al.* 2018). Additionally, here we found that the enzyme *GS* (6.3.1.2) in the GOGAT cycle that recycles waste ammonia into glutamine is significantly downregulated in bacteriocytes as the aphid ages in the 3rd *vs* 4th instar transition when feeding on fava (Figure 2). It has previously been hypothesized by Hansen and Moran (2011) that this cycle provides a key source of nitrogen for the biosynthesis of aphid and *Buchnera* provisioned amino acids inside of bacteriocytes. In support of this hypothesis, when aphids fed on a host plant that was lower in EAA concentrations (alfalfa) the same enzyme, *GS* (6.3.1.2), was significantly upregulated compared to when it fed on a plant that was higher in EAAs (fava), suggesting that this enzyme is important for recycling waste nitrogen to compensate for nitrogen limitation (Kim *et al.* 2018). These results suggest in combination that bacteriocytes of older nymphal and adult aphid lifestages depend less on the nutritional symbiosis when compared to early nymphal development lifestages where aphid bacteriocytes upregulate transcripts associated with EAA biosynthesis, NEAA transport, and recycling to supply *Buchnera* with aphid produced nitrogen and carbon metabolites.

Using two independent quantitative techniques for determining *Buchnera* titer, we found that *Buchnera* single gene copy count per aphid single gene copy count and *Buchnera* DNA reads mapped relative to aphid DNA reads mapped in bacteriocytes significantly decreased after the 1st instar, despite the aphid bacteriocyte numbers increasing throughout aphid development (Figure 1). Simonet *et al.* (2016) found the opposite trend for *Buchnera* titer throughout nymphal development using a new quantitative flow cytometry approach. However, this was an indirect measure of *Buchnera* titer in bacteriocytes because titer from aphid whole bodies was subtracted from embryos. A recent study conducted by Zhang *et al.* (2019), corroborates our data here and found that *Buchnera* titer decreases throughout nymphal development in *A. pisum*, in addition to several other aphid species, suggesting that this is a conserved pattern in aphid development.

Since *Buchnera* titer is known to vary depending on aphid genotype (Chong and Moran 2016) perhaps this developmental difference in *Buchnera* titer is due to aphid host regulation. Alternatively, *Buchnera* genotypes may also play a role in influencing their own titer. For example, several different *Buchnera* genotypes decrease in titer after 3–4 h of heat shock (35–37°C) due to a single basepair mutation they encode that decreases the expression of the heat shock gene *ibpA* (Dunbar *et al.* 2007; Moran and Yun 2015; Zhang *et al.* 2019). Higher temperatures can also increase *Buchnera* titer in young adult embryos positively when developing in a controlled temperature environment between 15°C and 25°C (Humphreys and Douglas 1997). Here we maintained genetically identical (clonal) sublines at 20°C for all trials and rearing conditions prior to trials so we do not anticipate that temperature variation or aphid genotype influenced *Buchnera* titer results here. Instead, we predict that a decrease in resources for *Buchnera* may explain the significantly lower titer after the 1st instar (Figure 1). Specifically, we predict that the aphid is metabolically regulating *Buchnera*’s productivity and production of EAA, vitamins, and titer by controlling *Buchnera*’s supply of host produced NEAAs (*e.g.* transporters and GOGAT cycle), glucose (*e.g.* via Tret-1), uracil, and other core metabolites that *Buchnera* cannot synthesize itself because aphid genes that produce these metabolites for *Buchnera* are significantly downregulated at intermediate and later nymphal lifestages compared to early nymphal stages in bacteriocytes (Figures 2–4). For example, here we observed the downregulation of Tret-1 during the 3rd to 4th instar transition for the transport of trehalose into bacteriocytes for the production of D-glucose. In turn, the aphid may reduce glycolysis to itself and its symbiont *Buchnera* by limiting the necessary precursors for energy production. Based on KEGG pathway enzyme enrichment patterns for aphid glycolysis/gluconeogenesis our results suggest that glycolysis is occurring in bacteriocytes during the early nymphal lifestages, and in corroboration with previous studies (Poliakov *et al.* 2011; Kim *et al.* 2018; Smith and Moran 2020), gluconeogenesis is potentially occurring during the intermediate and late nymphal lifestages and the young adult lifestage in bacteriocytes (2.7.1.40 and 2.7.1.11, but not 4.1.1.32 and 3.1.3.11, are enriched 1st and 2nd instars and depleted 4th instar and YA; Figures 3 and 4; Supplementary Datasets S4 and S5). In regard to uracil, enzymes that are enriched positively in the Beta-alanine pathway during the 2nd instar compared to other lifestages convert aspartate to beta-alanine, which is then converted into uracil and acetylCoA (4.1.1.15; 3.5.1.6; 1.3.1.2; 2.6.1.19; 1.2.1.18) (Supplementary Figure S6, Supplementary Datasets S4 and S5). In turn, we predict that uracil production peaks early on in nymphal development during the 2nd instar compared to later nymphal lifestages. Future studies are required to tease apart why *Buchnera* titer and aphid pathways for the metabolism of EAAs in bacteriocytes decrease as the aphid nymph ages.

*Buchnera* is hypothesized to synthesize methionine from homocysteine (Wilson *et al.* 2010). Methionine biosynthesis requires more energy (ATPs) compared to the biosynthesis of any other EAA in bacteria (Kaleta *et al.* 2013), and this amino acid has been highlighted as one of the most limited for plant feeding insects (Broderick and Strong 1987). Therefore, we predict that the aphid cystathionase-like enzyme (4.4.1.1), which collaborates with *Buchnera* in the production of methionine is tightly regulated throughout mid-late nymphal aphid development to reduce high energy costs of this nutritional symbiosis (Figure 2). For example, here we found that the cystathionase-like enzyme (4.4.1.1) is downregulated as the aphid ages for the sequential life stage comparisons 2nd *vs* 3rd and 3rd *vs* 4th (Figure 2, Table 2, Supplementary Table S3, Supplementary Dataset S6). This type of host-specific regulation may be wide-spread in other sap-feeding nutritional symbioses, because most sap-feeding endosymbionts have lost this intermediate step and potentially rely on the host for methionine biosynthesis (Hansen and Moran 2014). Alternatively, the aphid enzyme (4.4.1.1) may not be important for methionine biosynthesis. For example, for 7-day-old nymph bacteriocytes (potentially 4th instar), Russell *et al.* (2013) determined that the precursor cystathionine but not cysteine resulted in methionine production, suggesting that enzyme 4.4.1.1 does not synthesize cystathionine from cysteine. However, Russell *et al.* (2013) potentially did not observe the precursor cysteine promoting methionine production in 7-day nymphs because 4.4.1.1 is downregulated in bacteriocytes as the aphid ages in 2nd *vs* 3rd instars and 3rd *vs* 4th instars (Figure 2). Even though Russell *et al.* (2013) observed methionine production from the precursor cystathionine they could not identify the protein (4.2.1.22; LOC 100166111) being expressed from a previous study on 4th instar bacteriocytes (Poliakov *et al.* 2011). Here we suggest that an alternative enzyme 4.4.1.13 (Figure 2) (*e.g.* LOC100164839 or LOC100160464) based on KEGG pathways and gene expression analyses is responsible for synthesizing homocysteine from cystathionine. Both LOC100164839 and LOC100160464 are highly expressed for all life stages in bacteriocytes (between the 75th and 100% quartile based on normalized gene counts for all genes in all lifestages; Supplementary Dataset S1).

Tyrosine is important for insects for both the hardening of the insect’s exoskeleton (sclerotization) and the production of melanin for the insect’s innate immune response (Vavricka *et al.* 2014). Data here on aphid gene expression from all nymphal and adult lifestages suggest that the catabolism and biosynthesis of tyrosine and phenylalanine in aphid bacteriocytes are primarily important in 1st and 2nd instars compared to later lifestages when feeding on fava during nymphal development (Figures 3 and 4; Supplementary Dataset S4). Additionally, here we found that the production of DOPA from tyrosine via the enzyme tyrosine 3-monooxygenase (1.14.16.2), which is a rate-limited step, is downregulated as the aphid bacteriocyte ages in the 3rd *vs* 4th lifestage transition and further in the 4th instar *vs* young adult lifestage transition (Figure 2). This progressive downregulation of DOPA as the aphid ages suggests that the catabolism of tyrosine for sclerotization and melanization (Vavricka *et al.* 2014) is more important for earlier nymphal lifestages (1st to 2nd) of aphid development. In a recent study that examined aphid bacteriocyte gene expression patterns on artificial diets, aphids were fed on a diet deplete of the EAAs, tyrosine and phenylalanine, compared to a control diet (Colella *et al.* 2018). In this study, the authors observed transcriptional changes in the aphid bacteriocytes in response to tyrosine and phenylalanine depletion, including the downregulation of sugar transport and metabolism, and the upregulation of transcripts related to glycolysis, cell proliferation and size, and signaling. Colella *et al.* (2018) also found that there was a reduction in aphid weight for aphids feeding on the depleted diet compared to controls. They also observed increases in bacteriocyte number, size, and a 2-day delay in nymph laying between diet treatments. The delay in nymph laying and reduction in weight may mean that there was a significant difference in developmental rates between diet treatments. In turn, it is hard to dissect whether aphid gene expression differences that were observed at uniform 24 h/48 h time intervals for both treatments were due to diet, developmental differences, or both. For example, recently, it was observed that pea aphid growth rates vary dramatically when fed on different nutritional diets (Pers and Hansen 2019), further suggesting that studies are still needed that compare different diet treatments and the same lifestages. In another aphid transcriptome study, which focused on the embryonic lifestage and the first instar bacteriocyte, Rabatel *et al.* (2013) observed that the concentration of tyrosine increased dramatically throughout *A. pisum* embryonic development compared to other free amino acids, and then decreased rapidly within several hours of the early to late 1st instar lifestage when feeding on a plant diet (fava). Rabatel and colleagues (2013) suggest that the catabolism of tyrosine for cuticle formation is very important in the late embryonic lifestage compared to earlier embryonic stages or the 1st instar bacteriocyte (other nymphal or adult stages were not examined).

Numerous developmental genes and signaling pathways were dynamically enriched and depleted early in nymphal development (Supplementary Figures S4 and S7, Supplementary Table S4, Supplementary Datasets S4–S6). These pathways may play a role in establishing processes early on in nymphal bacteriocyte development, shutting off embryogenesis processes, and/or are involved in the regulation of the early nymphal metabolism in bacteriocytes. For example, given that the 1st instar is the first time the aphid is exposed to the environment and is no longer protected within its mother, an enrichment in phototransduction, neuroactive ligand, and Extracellular matrix (ECM)-receptor interactions may be expected as the aphid begins to interpret and respond to the surrounding environmental stimuli (Valmalette *et al.* 2012). Similarly, an upregulation of Toll and Imd pathways maybe expected since the innate immune system is vital for an organism being exposed to numerous, novel, and potentially toxic factors in the external environment.

The largest amount of signaling pathways was enriched in the 3rd instar stage compared to other lifestages (Supplementary Figure S7). Interestingly, Smith and Moran (2020) identified the same signaling pathways that were positively enriched here in the 3rd instar (Wnt, Notch, Hippo, Hedgehog, and TGF-beta) as upregulated in 4th instar bacteriocytes of low-*Buchnera* titer aphid genotypes, which focused primarily on aphid proliferation over nutrient/energy metabolism. These results collectively suggest that these signaling pathways may play a role in regulating aphid bacteriocyte cell maintenance, repair, and recycling processes, which is associated with a lower titer of *Buchnera* cells. For example, Wnt signaling is canonically known for cell-to-cell communication and noncanonically for cytoskeletal cellular polarity and cellular calcium levels (Komiya and Habas 2008). While Notch signaling is important for embryogenesis and neurogenesis (Bray 2016), and TGF signaling is important for cell growth, differentiation, regeneration, and death (Massague 2012). Similar to Simonet *et al.* (2016), our bacteriocyte count per aphid data demonstrate that the number of bacteriocyte cells continues to increase past embryogenesis (Figure 1, A and B). This suggests that bacteriocytes are dividing throughout nymphal development. Therefore, these pathways, most of which do not peak until the 3rd instar (Supplementary Figure S7) may be important in the nascent bacteriocytes. For example, we hypothesize that TGF signaling could be regulating the growth and differentiation of more mature bacteriocytes and Wnt signaling may be regulating the communication between bacteriocytes, body, and *Buchnera* cells in order to regulate a balance between energy demands and metabolite production.

To our knowledge, this is the first study to show that hox, noncanonical homeodomain, and signaling factor genes are differentially methylated during invertebrate development. For example, many vertebrate genes, especially hox genes and signaling factors (FGF, Hedgehog, NOTCH, TGF-β, WNT), undergo a widespread phase of de- and re-methylation during embryogenesis. This epigenetic reprogramming is thought to assist in the regulation of the spatiotemporal expression of vertebrate hox genes and the overall patterning of the embryo. However, invertebrates hox gene clusters and signaling factors are generally unmethylated and exhibit little change in methylation patterns within embryonic, gamete, and somatic tissues, suggesting methylation has no regulatory role in invertebrate patterning (Xu *et al.* 2019). However, in contrast to the honeybee and other invertebrates (Xu *et al.* 2019), we find one canonical hox genes (*Ubx*), eight noncanonical homeodomain-containing genes, and dozens of signaling factors are differentially methylated within bacteriocytes during aphid larvalgenesis (Supplementary Dataset S6). Additionally, three of these differentially methylated hox genes and four of these differentially methylated signal factors are also differentially expressed during the same life stage transition or the following lifestage transition (Supplementary Dataset S6). Hypermethylation may have a repressive role in six of these seven instances (a change in methylation followed by a change in gene expression in the opposite directions), suggesting, unlike other invertebrates, hox genes and signaling factors in aphid tissues are not only methylated and epigenetically reprogrammed, but also methylation may play a regulatory role in bacteriocytes during nymphal development. A role in symbiotic regulation is supported by findings that *Ubx* is required for bacteriocyte development in hemipterans (Matsuura *et al.* 2015), and both *Ubx* and *abdA* were novelty expressed in the ant tribe Camponotini, and co-opted to allow for the development of its symbiosis with Blochmannia (Rafiqi *et al.* 2020). These important developmental genes and their potential epigenetic regulation may play a key role in bacteriocyte development and the regulation of the aphid-*Buchnera* symbiosis. However, further studies are needed to determine if and how epigenetics plays a regulatory role in this symbiosis.

It is unclear why DNA methylation in bacteriocytes is more heterogenous for the 1st and 2nd instars compared to older lifestages. At the 1st and 2nd instars, there are significantly less bacteriocyte cells compared to later lifestages (1.8X and 1.3X less, respectively, compared to the 3rd instar; Figure 1A, Supplementary Table S1). In turn, potentially bacteriocytes that divide after second instar are largely homogenous in methylation profiles because there is a reduction in *de novo* methylation after 2nd instar. Consistent with this hypothesis, *DNMT3A* was enriched in the 2nd instar bacteriocytes and depleted in the young adult lifestage compared to other stages (Supplementary Dataset S5). A previous study observed that 4th instar bacteriocytes of *A. pisum* display host plant-specific DNA methylation profiles when aphids feed on two different host plants that vary in EAA concentrations (Kim *et al.* 2018). Based on data presented here and in Kim *et al.* (2018), we predict that host plant-specific DNA methylation profiles in bacteriocytes are determined early in aphid nymphal development (1st and 2nd instar) when the nymph begins to feed on the host plant that its mother larviposits it on. Aphids are largely sessile like other plant feeding hemipterans and remain on the same host plant and plant structure, which they initially settle on throughout nymphal development (Legrand and Barbosa 2000). It is of interest for future studies to determine if these *de novo* DNA methylation changes that occur and establish across nymphal development in bacteriocytes results in an adaptive or alternatively maladaptive host plant-specific response for the integrative aphid-symbiont nutritional metabolism. However, it is still unclear if any of these dynamic DNA methylation patterns in bacteriocytes are influencing gene expression patterns. While we identified 112 genes across nymphal developmental that were both differentially methylated and expressed within the same sequential lifestage comparison, 37% were hypomethylated and downregulated, 40% were hypermethylated and downregulated, 11% were hypomethylated and upregulated, and 12% were hypermethylated and upregulated (Supplementary Tables S3 and S4, Supplementary Dataset S6). Thus, overall, we found no correlation between the directional change of gene expression (up/downregulated) and the type of methylation change (*e.g.* hypo/hyper).

## Conclusions

Overall, based on our data, we predict that the aphid bacteriocyte is metabolically more active, especially in metabolizing *Buchnera*’s EAAs and vitamins (especially B vitamins), early in nymphal development (1st and 2nd) compared to intermediate or later lifestages that are generally analyzed for the aphid-*Buchnera* symbiosis (Hansen and Moran 2011; Poliakov *et al.* 2011; Moran and Yun 2015; Kim *et al.* 2018; Chung *et al.* 2018; Smith and Moran 2020). In turn, we hypothesize that the aphid demand for *Buchnera* synthesized EAAs and vitamins is highest in earlier lifestages of the aphid bacteriocyte. Potentially *Buchnera* derived nutrients are then exported from bacteriocytes and stored in other aphid tissues for the aphid’s energy and nutritional demands after the 2nd–3rd instar, because of the significant reduction in the expression of bacteriocyte transporters for carbon and nitrogen metabolites as the aphid ages at the 3rd to 4th lifestage transition. Following this potential shut down of the “amino acid factory,” we predict that the aphid bacteriocyte transitions from a “nutrient-consuming state” to a “growth and stress response/detoxification state,” demonstrated by the shift from an enrichment of nutrient-consuming and producing pathways that collaborate with *Buchnera* to an enrichment of information processing and cell signaling pathways, and the simultaneous increase in bacteriocyte cells and a reduction of *Buchnera* titer (Figures 1–4). Future studies that manipulate aphid DNA methylation, diet, and other environmental factors throughout nymphal development will further our understanding of the aphid-*Buchnera* symbiosis.

## References

[jkab115-B1] Ahuja N , Issa JP. 2000. Aging, methylation, and cancer. Histol Histopathol. 15:835–842. 10963127 10.14670/HH-15.835

[jkab115-B2] Akalin A , Kormaksson M, Li S, Garrett-Bakelman FE, Figueroa ME, et al 2012. methylKit: a comprehensive R package for the analysis of genome-wide DNA methylation profiles. Genome Biol. 13:R87. 23034086 10.1186/gb-2012-13-10-r87 PMC3491415

[jkab115-B3] Andrews S. 2010. FastQC: a quality control tool for high throughput sequence data. (Accessed: 2011 October 6). http://www.bioinformatics.babraham.ac.uk?/projects/fastqc/.

[jkab115-B4] Awmack CS , Leather SR. 2002. Host plant quality and fecundity in herbivorous insects. Annu Rev Entomol. 47:817–844. 11729092 10.1146/annurev.ento.47.091201.145300

[jkab115-B5] Bolger AM , Lohse M, Usadel B. 2014. Trimmomatic: a flexible trimmer for Illumina sequence data. Bioinformatics. 30:2114–2120. 24695404 10.1093/bioinformatics/btu170 PMC4103590

[jkab115-B6] Braendle C , Miura T, Bickel R, Shingleton AW, Kambhampati S, et al 2003. Developmental origin and evolution of bacteriocytes in the aphid-*Buchnera* symbiosis. PLoS Biol. 1:e21. 14551917 10.1371/journal.pbio.0000021 PMC212699

[jkab115-B7] Bray SJ. 2016. Notch signaling in context. Nat Rev Mol Cell Biol. 17:722–735. 27507209 10.1038/nrm.2016.94

[jkab115-B8] Brisson JA. 2010. Aphid wing dimorphisms: linking environmental and genetic control of trait variation. Philos Trans R Soc Lond B Biol Sci. 365:605–616. 20083636 10.1098/rstb.2009.0255 PMC2817143

[jkab115-B9] Broderick B , Strong D. 1987. Amino acid nutrition of herbivorous insects and stress to host plants. In: Barbosa P, Schultz J, editors. Insect Outbreaks: ecological and Evolutionary Perspectives. New York, NY: Academic Press Inc. p. 347–364.

[jkab115-B10] Chong RA , Moran NA. 2016. Intraspecific genetic variation in hosts affects regulation of obligate heritable symbionts. Proc Natl Acad Sci U S A. 113:13114–13119. 27799532 10.1073/pnas.1610749113 PMC5135297

[jkab115-B11] Chung SH , Jing X, Luo Y, Douglas AE. 2018. Targeting symbiosis-related insect genes by RNAi in the pea aphid-*Buchnera* symbiosis. Insect Biochem Mol Biol. 95:55–63. 29526771 10.1016/j.ibmb.2018.02.004

[jkab115-B12] Colella S , Parisot N, Simonet P, Gaget K, Duport G, et al 2018. Bacteriocyte reprogramming to cope with nutritional stress in a phloem sap feeding hemipteran, the pea aphid *Acyrthosiphon pisum*. Front Physiol. 9:1498. 30410449 10.3389/fphys.2018.01498 PMC6209921

[jkab115-B13] Dixon AFG. 1977. Aphid ecology: life cycles, polymorphism, and population regulation. Annu Rev Ecol Syst. 8:329–353.

[jkab115-B14] Douglas AE , Dixon AFG. 1987. The mycetocyte symbiosis of aphids: variation with age and morph in virginoparae of *Megoura viciae* and *Acyrthosiphon pisum*. J Insect Physiol. 33:109–113.

[jkab115-B15] Dunbar HE , Wilson ACC, Ferguson NR, Moran NA. 2007. Aphid thermal tolerance is governed by a point mutation in bacterial symbionts. PLoS Biol. 5:e96. 17425405 10.1371/journal.pbio.0050096 PMC1847839

[jkab115-B16] Feng H , Edwards N, Anderson CMH, Althaus M, Duncan RP, et al 2019. Trading amino acids at the aphid-*Buchnera* symbiotic interface. Proc Natl Acad Sci U S A. 116:16003–16011. 31337682 10.1073/pnas.1906223116 PMC6690024

[jkab115-B17] Field LM. 2000. Methylation and expression of amplified esterase genes in the aphid *Myzus persicae* (Sulzer). Biochem J. 349:863–868. 10903149 10.1042/bj3490863 PMC1221215

[jkab115-B18] Flórez LV , Biedermann PHW, Engl T, Kaltenpoth M. 2015. Defensive symbioses of animals with prokaryotic and eukaryotic microorganisms. Nat Prod Rep. 32:904–936. 25891201 10.1039/c5np00010f

[jkab115-B19] Ginzburg N , Cohen M, Chipman AD. 2017. Factors involved in early polarization of the anterior-posterior axis in the milkweed bug *Oncopeltus fasciatus*. Genesis. 55:e23027. 10.1002/dvg.23027 28432817

[jkab115-B20] Glastad KM , Hunt BG, Yi SV, Goodisman MAD. 2011. DNA methylation in insects: on the brink of the epigenomic era. Insect Mol Biol. 20:553–565. 21699596 10.1111/j.1365-2583.2011.01092.x

[jkab115-B21] Hansen AK , Moran NA. 2011. Aphid genome expression reveals host-symbiont cooperation in the production of amino acids. Proc Natl Acad Sci U S A. 108:2849–2854. 21282658 10.1073/pnas.1013465108 PMC3041126

[jkab115-B22] Hansen AK , Moran NA. 2014. The impact of microbial symbionts on host plant utilization by herbivorous insects. Mol Ecol. 23:1473–1496. 23952067 10.1111/mec.12421

[jkab115-B23] Humphreys NJ , Douglas AE. 1997. Partitioning of symbiotic bacteria between generations of an insect, a quantitative study of a *Buchnera* sp. in the pea aphid (*Acyrthosiphon pisum*) reared at different temperatures. Appl Environ Microbiol. 63:3294–3296. 16535678 10.1128/aem.63.8.3294-3296.1997 PMC1389233

[jkab115-B24] Kaleta C , Schauble S, Rinas U, Schuster S. 2013. Metabolic costs of amino acid and protein production in *Escherichia coli*. Biotechnol J. 8:1105–1114. 23744758 10.1002/biot.201200267

[jkab115-B25] Kim D , Langmead B, Salzberg SL. 2015. HISAT: a fast spliced aligner with low memory requirements. Nat Methods. 12:357–360. 25751142 10.1038/nmeth.3317 PMC4655817

[jkab115-B26] Kim D , Minhas BF, Li-Byarlay H, Hansen AK. 2018. Key transport and ammonia recycling genes involved in aphid symbiosis respond to host-plant specialization. G3 (Bethesda). 8:2433–2443. 29769291 10.1534/g3.118.200297 PMC6027869

[jkab115-B27] Kim D , Song L, Breitwieser FP, Salzberg SL. 2016. Centrifuge: rapid and sensitive classification of metagenomic sequences. Genome Res. 26:1721–1729. 27852649 10.1101/gr.210641.116 PMC5131823

[jkab115-B28] Koga R , Meng X-Y, Tsuchida T, Fukatsu T. 2012. Cellular mechanism for selective vertical transmission of an obligate insect symbiont at the bacteriocyte-embryo interface. Proc Natl Acad Sci U S A. 109:E1230–E1237. 22517738 10.1073/pnas.1119212109 PMC3356617

[jkab115-B29] Komiya Y , Habas R. 2008. Wnt signal transduction pathways. Organogenesis. 4:68–75. 19279717 10.4161/org.4.2.5851 PMC2634250

[jkab115-B30] Krueger F , Andrews SR. 2011. Bismark: a flexible aligner and methylation caller for Bisulfite-Seq applications. Bioinformatics. 27:1571–1572. 21493656 10.1093/bioinformatics/btr167 PMC3102221

[jkab115-B31] Kucukural A , Yukselen O, Ozata DM, Moore MJ, Garber M. 2019. DEBrowser: interactive differential expression analysis and visualization tool for count data. BMC Genomics. 20:6. 30611200 10.1186/s12864-018-5362-x PMC6321710

[jkab115-B32] Legrand A , Barbosa P. 2000. Pea aphid (Homoptera: Aphididae) fecundity, rate of increase, and within-plant distribution unaffected by plant morphology. Environ Entomol. 29:987–993.

[jkab115-B33] Li Y , Park H, Smith TE, Moran NA. 2019. Gene family evolution in the pea aphid based on chromosome-level genome assembly. Mol Biol Evol. 36:2143–2156. 31173104 10.1093/molbev/msz138 PMC6759078

[jkab115-B34] Mallo M , Alonso CR. 2013. The regulation of Hox gene expression during animal development. Development. 140:3951–3963. 24046316 10.1242/dev.068346

[jkab115-B35] Massague J. 2012. TGFB signaling in context. Nat Rev Mol Cell Biol. 13:616–630. 22992590 10.1038/nrm3434 PMC4027049

[jkab115-B36] Mathers TC , Mugford ST, Percival-Alwyn L, Chen Y, Kaithakottil G, et al 2019. Sex-specific changes in the aphid DNA methyl ation landscape. Mol Ecol. 28:4228–4241. 31472081 10.1111/mec.15216 PMC6857007

[jkab115-B37] Matsuura Y , Kikuchi Y, Miura T, Fukatsu T. 2015. *Ultrabithorax* is essential for bacteriocyte development. Proc Natl Acad Sci U S A. 112:9376–9381. 26170303 10.1073/pnas.1503371112 PMC4522796

[jkab115-B38] McFall-Ngai M , Hadfield MG, Bosch TCG, Carey HV, Domazet-Loso T, et al 2013. Animals in a bacterial world, a new imperative for the life sciences. Proc Natl Acad Sci U S A. 110:3229–3236. 23391737 10.1073/pnas.1218525110 PMC3587249

[jkab115-B39] Miura T , Braendle C, Shingleton A, Sisk G, Kambhampati S, et al 2003. A comparison of parthenogenetic and sexual embryogenesis of the pea aphid *Acyrthosiphon pisum* (Hemiptera: Aphidoidea). J Exp Zool B Mol Dev Evol. 295:59–81. 12548543 10.1002/jez.b.3

[jkab115-B40] Moran NA , Yun Y. 2015. Experimental replacement of an obligate insect symbiont. Proc Natl Acad Sci U S A. 112:2093–2096. 25561531 10.1073/pnas.1420037112 PMC4343100

[jkab115-B41] Pasquier C , Clement M, Dombrovsky A, Penaud S, Da Rocha M, et al 2014. Environmentally selected aphid variants in clonality context display differential patterns of methylation in the genome. PLoS One. 9:e115022. 25551225 10.1371/journal.pone.0115022 PMC4281257

[jkab115-B42] Pers D , Hansen AK. 2019. The effects of different diets and transgenerational stress on *Acyrthosiphon pisum* development. Insects. 10:260. 10.3390/insects10090260 PMC6780513 31438654

[jkab115-B43] Pers D , Hansen AK. 2020. Life stage specific transcriptomes and methylomes from *A. pisum* bacteriocytes. NCBI-SRA; SRA accession: PRJNA613906. www.ncbi.nlm.nih.gov/sra/PRJNA613906.

[jkab115-B44] Pertea M , Kim D, Pertea GM, Leek JT, Salzberg SL. 2016. Transcript-level expression analysis of RNA-seq experiments with HISAT, StringTie and Ballgown. Nat Protoc. 11:1650–1667. 27560171 10.1038/nprot.2016.095 PMC5032908

[jkab115-B45] Pertea M , Pertea GM, Antonescu CM, Chang TC, Mendell JT, et al 2015. StringTie enables improved reconstruction of a transcriptome from RNA-seq reads. Nat Biotechnol. 33:290–295. 25690850 10.1038/nbt.3122 PMC4643835

[jkab115-B46] Poliakov A , Russell CW, Ponnala L, Hoops HJ, Sun Q, et al 2011. Large-scale label-free quantitative proteomics of the pea aphid-*Buchnera* symbiosis. Mol Cell Proteomics. 10:M110.007039. 10.1074/mcp.M110.007039 PMC3108839 21421797

[jkab115-B47] Price DRG , Feng H, Baker JD, Bavan S, Luetje CW, et al 2014. Aphid amino acid transporter regulates glutamine supply to intracellular bacterial symbionts. Proc Natl Acad Sci U S A. 111:320–325. 24367072 10.1073/pnas.1306068111 PMC3890774

[jkab115-B48] Rabatel A , Febvay G, Gaget K, Duport G, Baa-Puyoulet P, et al 2013. Tyrosine pathway regulation is host-mediated in the pea aphid symbiosis during late embryonic and early larval development. BMC Genomics. 14:235. 23575215 10.1186/1471-2164-14-235 PMC3660198

[jkab115-B49] Rafiqi AM , Rajakumar A, Abouheif E. 2020. Origin and elaboration of a major evolutionary transition in individuality. Nature. 585:239–244. 32879485 10.1038/s41586-020-2653-6

[jkab115-B50] Ramakers C , Ruijter JM, Lekanne Deprez RH, Moorman AFM. 2003. Assumption-free analysis of quantitative real-time polymerase chain reaction (PCR) data. Neurosci Lett. 339:62–66. 12618301 10.1016/s0304-3940(02)01423-4

[jkab115-B51] Richardson B. 2003. Impact of aging on DNA methylation. Ageing Res Rev. 2:245–261. 12726774 10.1016/s1568-1637(03)00010-2

[jkab115-B52] Robinson MD , McCarthy DJ, Smyth GK. 2010. edgeR: a Bioconductor package for differential expression analysis of digital gene expression data. Bioinformatics. 26:139–140. 19910308 10.1093/bioinformatics/btp616 PMC2796818

[jkab115-B53] Romanoski CE , Glass CK, Stunnenberg HG, Wilson L, Almouzni G. 2015. Roadmap for regulation. Nature. 518:314–316. 25693562 10.1038/518314a

[jkab115-B54] Ruijter JM , Ramakers C, Hoogaars WMH, Karlen Y, Bakker O, et al 2009. Amplification efficiency: linking baseline and bias in the analysis of quantitative PCR data. Nucleic Acids Res. 37:e45. 19237396 10.1093/nar/gkp045 PMC2665230

[jkab115-B55] Russell CW , Bouvaine S, Newell PD, Douglas AE. 2013. Shared metabolic pathways in a coevolved inset-bacterial symbiosis. Appl Environ Microbiol. 79:6117–6123. 23892755 10.1128/AEM.01543-13 PMC3811349

[jkab115-B56] Shigenobu S , Bickel RD, Brisson JA, Butts T, Chang C-C, et al 2010. Comprehensive survey of developmental genes in the pea aphid, *Acyrthosiphon pisum*: frequent lineage‐specific duplications and losses of developmental genes. Insect Mol Biol. 19:47–62. 20482639 10.1111/j.1365-2583.2009.00944.x

[jkab115-B57] Shigenobu S , Wilson ACC. 2011. Genomic revelations of a mutualism: the pea aphid and its obligate bacterial symbiont. Cell Mol Life Sci. 68:1297–1309. 21390549 10.1007/s00018-011-0645-2 PMC3064905

[jkab115-B58] Simonet P , Duport G, Gaget K, Weiss-Gayet M, Colella S, et al 2016. Direct flow cytometry measurements reveal a fine-tuning of symbiotic cell dynamics according to the host developmental needs in aphid symbiosis. Sci Rep. 6:19967. 26822159 10.1038/srep19967 PMC4731799

[jkab115-B59] Skidmore IH , Hansen AK. 2017. The evolutionary development of plant‐feeding insects and their nutritional endosymbionts. Insect Sci. 24:910–928. 28371395 10.1111/1744-7917.12463

[jkab115-B60] Smith TE , Moran NA. 2020. Coordination of host and symbiont gene expression reveals a metabolic tug-of-war between aphids and *Buchnera*. Proc Natl Acad Sci U S A. 117:2113–2121. 31964845 10.1073/pnas.1916748117 PMC6995025

[jkab115-B61] Srinivasan DG , Brisson JA. 2012. Aphids: a model for polyphenism and epigenetics. Genet Res Int. 2012:431531. 22567389 10.1155/2012/431531 PMC3335499

[jkab115-B62] Stockhoff BA. 1993. Ontogenic change in dietary selection for protein and lipid by gypsy moth larvae. J Insect Physiol. 39:677–686.

[jkab115-B63] Subramanian A , Tamayo P, Mootha VK, Mukherjee S, Ebert BL, et al 2005. Gene set enrichment analysis: a knowledge-based approach for interpreting genome-wide expression profiles. Proc Natl Acad Sci U S A. 102:15545–15550. 16199517 10.1073/pnas.0506580102 PMC1239896

[jkab115-B64] Valmalette JC , Dombrovsky A, Brat P, Mertz C, Capovilla M, et al 2012. Light- induced electron transfer and ATP synthesis in a carotene synthesizing insect. Sci Rep. 2:579. 22900140 10.1038/srep00579 PMC3420219

[jkab115-B65] Vavricka CJ , Han Q, Mehere P, Ding H, Christensen BM, et al 2014. Tyrosine metabolic enzymes from insects and mammals: a comparative perspective. Insect Sci. 21:13–19. 23955993 10.1111/1744-7917.12038

[jkab115-B66] Walsh TK , Brisson JA, Robertson HM, Gordon K, Jaubert-Possami S, et al 2010. A functional DNA methylation system in the pea aphid, *Acyrthosiphon pisum*. Insect Mol Biol. 19:215–228. 20482652 10.1111/j.1365-2583.2009.00974.x

[jkab115-B67] Wigglesworth VB. 1960. Nutrition and reproduction in insects. Proc Nutr Soc. 19:18–23. 13844694 10.1079/pns19600007

[jkab115-B68] Wilson ACC , Ashton PD, Calevro F, Charles H, Colella S, Febvay G, et al 2010. Genomic insight into the amino acid relations of the pea aphid, *Acyrthosiphon pisum*, with its symbiotic bacterium *Buchnera aphidicola*. Insect Mol Biol. 19:249–258. 20482655 10.1111/j.1365-2583.2009.00942.x

[jkab115-B69] Xu X , Li G, Li C, Zhang J, Wang Q, et al 2019. Evolutionary transition between invertebrates and vertebrates via methylation reprogramming in embryogenesis. Natl Sci Rev. 6:993–1003. 10.1093/nsr/nwz064 PMC8291442 34691960

[jkab115-B70] Yang K , Chen Y, Zhang Z, Kwok-Shing Ng P, Zhou WJ, et al 2016. Transcriptome analysis of different developmental stages of amphioxus reveals dynamic changes of distinct classes of genes during development. Sci Rep. 6:23195. 26979494 10.1038/srep23195 PMC4793263

[jkab115-B71] Zhang B , Leonard SP, Li Y, Moran NA. 2019. Obligate bacterial endosymbionts limit thermal tolerance of insect host species. Proc Natl Acad Sci U S A. 116:24712–24718. 31740601 10.1073/pnas.1915307116 PMC6900525

[jkab115-B72] Ziller MJ , Hansen KD, Meissner A, Aryee MJ. 2015. Coverage recommendations for methylation analysis by whole-genome bisulfite sequencing. Nat Methods. 12:230–232. 25362363 10.1038/nmeth.3152 PMC4344394

